# Effects of neck-specific exercise with or without a behavioural approach in addition to prescribed physical activity for individuals with chronic whiplash-associated disorders: a prospective randomised study

**DOI:** 10.1186/1471-2474-14-311

**Published:** 2013-10-30

**Authors:** Anneli Peolsson, Maria Landén Ludvigsson, Thomas Overmeer, Åsa Dedering, Lars Bernfort, Gun Johansson, Ann-Sofi Kammerlind, Gunnel Peterson

**Affiliations:** 1 Department of Medical and Health Sciences, Physiotherapy, Faculty of Health Sciences, Linköping University, Linköping, Sweden; 2 NHMRC CCRE (Spinal Pain, Injury and Health), The University of Queensland, Brisbane, Australia; 3 Rehab Väst, County Council of Östergötland, Motala, Sweden; 4 School of Health, Care and Social Welfare, Mälardalen University, Västerås, Sweden; 5 Center for Health and Medical Physiology, Örebro University, Örebro, Sweden; 6 Department of Physical Therapy, Karolinska University Hospital, Stockholm, Sweden; 7 Department of Neurobiology, Care Sciences and Society, Division of Physiotherapy, Karolinska Institutet, Stockholm, Sweden; 8 Department of Medical and Health Sciences, Health Care Analysis, Linköping University, Linköping, Sweden; 9 Department of Medical and Health Sciences, Community Medicine, Linköping University, Linköping, Sweden; 10 Futurum the academy for Healthcare, Jönköping, Sweden; 11 County Council of Jönköping, Jönköping, Sweden; 12 Centre for Clinical Research Sörmland, Uppsala University, Uppsala, Sweden

**Keywords:** Whiplash injuries, Neck pain, Spine, Rehabilitation, Physical therapy, Exercise

## Abstract

**Background:**

Up to 50% of chronic whiplash associated disorders (WAD) patients experience considerable pain and disability and remain on sick-leave. No evidence supports the use of physiotherapy treatment of chronic WAD, although exercise is recommended. Previous randomised controlled studies did not evaluate the value of adding a behavioural therapy intervention to neck-specific exercises, nor did they compare these treatments to prescription of general physical activity. Few exercise studies focus on patients with chronic WAD, and few have looked at patients’ ability to return to work and the cost-effectiveness of treatments. Thus, there is a great need to develop successful evidence-based rehabilitation models. The study aim is to investigate whether neck-specific exercise with or without a behavioural approach (facilitated by a single caregiver per patient) improves functioning compared to prescription of general physical activity for individuals with chronic WAD.

**Methods/Design:**

The study is a prospective, randomised, controlled, multi-centre study with a 2-year follow-up that includes 216 patients with chronic WAD (> 6 months and < 3 years). The patients (aged 18 to 63) must be classified as WAD grade 2 or 3. Eligibility will be determined with a questionnaire, telephone interview and clinical examination. The participants will be randomised into one of three treatments: (A) neck-specific exercise followed by prescription of physical activity; (B) neck-specific exercise with a behavioural approach followed by prescription of physical activity; or (C) prescription of physical activity alone without neck-specific exercises. Treatments will be performed for 3 months. We will examine physical and psychological function, pain intensity, health care consumption, the ability to resume work and economic health benefits. An independent, blinded investigator will perform the measurements at baseline and 3, 6, 12 and 24 months after inclusion. The main study outcome will be improvement in neck-specific disability as measured with the Neck Disability Index. All treatments will be recorded in treatment diaries and medical records.

**Discussion:**

The study findings will help improve the treatment of patients with chronic WAD.

**Trials registration:**

ClinicalTrials.gov identifier: NCT01528579.

## Background

Whiplash associated disorders (WAD) are common [1-4], with a cumulative annual incidence as high as 600 per 100 000 inhabitants [5]. When symptoms persist for more than 6 months, WAD is considered chronic [6]. Recent studies estimate that up to 50% of WAD patients experience prolonged symptoms, including considerable pain, disability, sick leave and reduced income [7-9].

Due to the personal and societal burdens associated with chronic WAD [1,2,9], it is extremely important to determine the best possible treatment for reducing pain and restoring the ability of patients to perform everyday tasks and to return to work. A WAD diagnosis is based on a patient’s subjective description of symptoms and on clinical examination. The Quebec Task Force (QTF) classified WAD into severity grades 0–4, with grade 0 indicating no neck complaints and no physical sign(s) and grade 4 indicating a neck complaint with neck fracture or dislocation [10]. Identifying subgroups in this heterogeneous group of patients with chronic WAD could lead to a better understanding of the complexity of chronic WAD [11].

There is no consensus regarding the injury mechanism in complex WAD cases. Some symptoms of persistent WAD can be attributed to injuries involving neck structures, including joints, ligaments and muscles [12]. In particular, WAD may involve an altered activation pattern in the neck muscles [13]. Combinations of bio- and psychosocial factors, such as fear of re-injury, low mood and low self-efficacy, appear to affect recovery [14,15]. Thus, it is reasonable to assume that a combination treatment that includes both a physical and a behavioural approach might improve rehabilitative outcomes [16]. However, it is time-consuming and costly to involve several professionals in an individual’s treatment. Unfortunately, little is known about how a single caregiver can best use a combined physical and behavioural approach to treat individuals with chronic WAD [16,17]. Specifically, the effect of adding a behavioural component to neck-specific exercises is unknown. Furthermore, few studies on individuals with chronic WAD symptoms have investigated the effects of exercises that specifically target neck muscles.

Most studies of people with WAD have focused on the acute phase. The general advice given to patients in this phase of WAD is to be physically active [1,18]. There are just a few randomised controlled trials (RCTs) of the effects on exercise on chronic WAD [14,15,19-21]. Of these, two studies had small samples [15,19], and two other studies [20,21] allowed the addition of passive treatment strategies, making it difficult to determine the effects of the different treatment components. Only one larger RCT, conducted by Stewart et al. [14], included patients with WAD grade 3 and compared a 6-week exercise and behavioural programme to self-treatment based on professional advice. Pain intensity, pain bothersomeness and function were used as primary outcome measurements, and the short-term outcome favoured professional advice in combination with exercise over advice alone.

In all of the WAD RCTs to date, the effects of treatment have been limited [17]. Unfortunately, there is a lack of knowledge of how exercise regimens should be designed to successfully treat chronic WAD [11]. No evidence is available that supports physiotherapy treatment of chronic WAD [17], although exercises are recommended [22]. There are no studies that compare the effect of physical activity, which is commonly prescribed [18], with other approaches, such as neck-specific exercises or a behavioural intervention performed by a physiotherapist. No studies have investigated the outcomes of treatment in terms of the ability of patients to resume work or the cost-effectiveness of different exercise programmes with or without behavioural therapy. Thus, there is a great need for information about which rehabilitation models are effective and cost-efficient.

The aim of this study is to investigate whether neck-specific exercise with or without a behavioural component (facilitated by a single caregiver per patient) improves functioning compared to prescription of general physical activity for individuals with chronic WAD.

## Methods/Design

### Study population

The study will include 216 patients with WAD that has lasted more than 6 months but less than 3 years. The included patients will be randomised into one of the three treatment arms.

#### Eligibility criteria

The inclusion criteria are: Age 18–63 years; WAD grade 2–3 after a whiplash injury at least six months but not more than three years ago, QTF grade 2 includes neck complaints and musculoskeletal sign(s), grade 3 includes grade 2 plus neurological sign(s); Pain intensity > 20 mm on a 100-mm Visual Analogue Scale (VAS) [23] and/or > 20% on the Neck Disability Index (NDI, 0–100%) [24].

Exclusion criteria are: Known or suspected serious physical pathology, including myelopathy, spinal tumour, spinal infection or ongoing malignancy; Earlier fracture or luxation of the cervical column; Neck trauma with persistent symptoms from previous injury; Surgery on the cervical column; Neck pain that caused a > 1 month absence from work in the year prior to the WAD trauma; Signs of traumatic brain injury at the time of WAD (unconsciousness, retrograde or post-traumatic amnesia, disorientation or confusion); Generalised or more dominant pain elsewhere in the body; Diseases or other injuries that might prevent full participation in the study; Diagnosis of a severe psychiatric disorder; Known drug abuse; Insufficient knowledge of the Swedish language (inability to answer the questionnaires).

### Design

This study is a prospective, randomised, controlled, multi-centre study with a 2-year follow-up. Patients will be recruited by searching electronic medical records in five counties in Sweden. Individuals that sought care in the previous 6 to 36 months due to WAD will receive written information that describes the study and that asks whether they are interested in participating. Interested individuals will be screened via questionnaires and telephone interviews by an experienced physiotherapist to determine eligibility and to provide standardised oral information about the study (with a neutral description of the treatment alternatives). Eligible individuals will be asked to undergo a physical examination to ensure that their symptoms are consistent with WAD grade 2 or 3. After providing informed consent, eligible patients will be included in the study. The estimated time period for patient inclusion into the study is 2 years. Next, the patients will be randomised to receive one of three alternative physiotherapy treatments: (A) neck-specific exercise followed by prescription of physical activity; (B) neck-specific exercise with a behavioural approach followed by prescription of physical activity; or (C) prescription of physical activity alone without neck-specific exercises. Treatment will be performed by physiotherapists in primary care centres or private outpatient clinics. The physiotherapists will receive oral and written information about the treatment programs and will participate in an educational session and practice the different interventions. All treatments will be recorded in a diary and in medical records.

Randomisation will be based on a computer-generated list created by the university statistician. This list will assign patient ID numbers to group A, B or C and will be managed by an independent researcher who is not involved in the study in any way. Data from the randomised individuals will be distributed into sequentially numbered, sealed, opaque envelopes. Each envelope will be sent to the treating physiotherapist (in primary care centres or at private outpatient clinics) who will open the envelope and make an appointment with the identified participant. Due to the nature of the treatment, it will not be possible to blind the physiotherapists or the participants to the treatment.

### Outcome measurements

The measurements (clinical measurements and questionnaires) will be collected before treatment and 3, 6 and 12 months after inclusion. Questionnaires will also be collected at a 24-month follow-up. Clinical (neck-related) measurements will be performed in a standardised manner by a well-trained independent investigator who is blinded to the randomisation procedure and who is not involved in the interventions. Independent investigators will also collect the questionnaires. The questionnaires will ask about the patient’s background (including age, gender, social status, education, smoking, pain duration, previous treatment, information about the whiplash trauma and other medical conditions), and about disease-specific information and generic data. The main treatment outcome is neck-specific disability as measured with the NDI [24].

#### Clinical measurements

Clinical measurements includes: Neurological assessment: sensibility, motor function, reflexes, Spurling’s test, neuro-dynamic tests; Active range of motion of the neck in three planes [25] and cervical kinaesthesia (the ability to reproduce the neutral head position from 30° cervical rotation with the eyes closed) [26] as measured with a plastic helmet known as a “cervical range of motion device” [27]; Anterior and posterior neck muscle endurance in seconds in a supine and prone position [28]; Isometric hand grip strength as measured with the Jamar Hand Dynamometer [29]; Static and dynamic clinical balance: static clinical balance as assessed with the Sharpened Romberg test, with eyes closed and the non-dominant foot in front of the dominant foot [30]; dynamic clinical balance as assessed by the patient walking in a figure eight [31].

#### Questionnaires

Primary outcome measure is Neck-specific function: measured with the NDI [24].

Secondery outcome measures contained questionnaires about: Neck pain intensity/ bothersomeness; Pain intensity in the head and arm; Dizziness and unsteadiness: measured with a Visual Analogue Scale (VAS) (0–100 mm) [23]; Domain-specific and general disability related to chronic pain measured with the Pain Disability Index [32]; Dizziness: measured with the UCLA Dizziness Questionnaire [33]; Frequency of symptoms, including neck pain, arm pain, headache, dizziness, visual disturbances, tinnitus, difficulty swallowing and problems concentrating: measured with a 5-grade scale; Pain catastrophising: measured with the Pain Catastrophising Scale [34]; Ability to perform important activities, self-rated by the patient: measured with the Patient-Specific Functional Scale and recorded by the physiotherapist [35]; Confidence in the ability to perform different activities: measured with the Self-Efficacy Scale [36]; Confidence in the ability to perform physical training: measured with the Exercise Self-Efficacy Scale [37]; Physical activity: estimated with the International Physical Activity Questionnaire [38]; Operating fear: measured with the Tampa Scale of Kinesiophobia, short version [39]; Depression and anxiety: measured with the Hospital Anxiety and Depression Scale [40]; Health-related quality of life: measured with the EuroQol-five dimension questionnaire [40], EuroQol thermometer (0–100) [41] and SF-36 [42]; Work conditions, including type of work, work ability and physical demands at work: measured with the Work Ability Index [43]; work-related stress: measured with the Effort Reward Imbalance at work [44]; work satisfaction: measured with various specific questions; The number of sick leave days and extent and disability pension data (collected from the Swedish Social Insurance Agency), the number of sick leave days is also answered on the questionnaire, health care consumption measured with questions about type of caregiver and number of visits.

Questionnaire to determine fulfilment and satisfaction after treatment (to be filled in only after the treatment is completed) are: Treatment success regarding the difference in function after treatment (on a 6-grade scale) and the importance of the difference (0–100-mm VAS scale); Fulfilment of treatment expectations (on a 3-point scale); Satisfaction with the information and care provided measured with the Patient Enablement Instrument (PEI) [45] and an open question.

#### Cost-effectiveness determination

Direct costs, mainly health care costs, including the quantity and type of health care visits: determined from questionnaires and from patient interviews. Indirect costs, mainly production loss (i.e., inability to perform work due to ill health). According to economic theory, production loss is calculated as the gross income plus taxes for the time period that the patient is absent from work. Sick-leave compensation and income data will be collected from the Social Insurance Office. The effectiveness of interventions will be evaluated in terms of quality-adjusted life years (QALYs). Cost-effectiveness will be evaluated by comparing the costs and effects of the three interventions.

### Interventions

All therapists will be required to keep records of the exercises performed, the behavioural interventions given and the progress of each exercise in a diary.

#### Group A. Neck-specific exercise followed by prescription of physical activity

Neck-specific exercises will be performed twice weekly at the physiotherapy clinic and daily at home. The exercise therapy focuses on re-learning motor skills, neck muscle endurance and postural correction. These exercises aim to increase coordination, endurance and the strength of the muscles that stabilise the neck and scapula. After the clinical examination, the experienced physiotherapist will design an exercise programme from a well-defined set of exercises with a standardised, structured progression plan. The physiotherapist will adjust the programme for each patient to ensure that the selection of exercise and dosages are appropriate for the participant’s ability. The exercises will be performed daily at home. The exercises are not supposed to cause pain.

After 3 months, the participant will receive a printed individualized prescription for physical activity similar to that provided to group C (see below), but which includes neck-specific exercises. The aim is to encourage the patient to continue exercising to maintain and further improve their functioning without depending on a therapist.

#### Group B. Neck-specific exercise with a behavioural approach followed by prescription of physical activity

Each participant will undergo a three-month behavioural approach in combination with neck-specific exercises; the approach and exercise will be supervised by the physiotherapist at the physiotherapy clinic (twice weekly). The exercises are the same as those in group A, but with the addition of a behavioural component. Patients will listen to lectures about the basic neuroscience of pain. The physiotherapist will help the participants set realistic and specific activity goals, the aim of which is improvement of daily function. The physiotherapist and patient will discuss beliefs and barriers to recovery and methods for managing symptom relapse. This aims to decrease fear/avoidance and to increase self-efficacy and patient willingness to perform physical activity, despite the pain. It will also teach the patient how to best deal with the pain and provide greater understanding of the relationship between thoughts, emotions, body function and WAD symptoms. Participants will also be taught home exercises that focus on using relaxation to reduce stress and muscle tension and on postural correction through body awareness techniques. Additional home exercises (directed towards the participants’ activity goals) will be performed as well. After 3 months, the participant will receive a printed individualized prescription for physical activity, including neck-specific exercises like those provided to group A.

#### Group C. Prescription of physical activity alone

Each participant will have one or two appointments to complete a physical examination and a short motivational interview at the physiotherapy clinic. The interview will include determining the patient’s willingness and motivation to adopt new exercise routines and provide information about the benefits of physical activity. The participant will receive a printed copy of individualised, accessible, physical activity instructions that do not include neck-specific exercise. The aim of these activities is to increase the general level of physical activity (i.e. to improve fitness and to elicit the release of endorphins). The activity can be performed in a selected location outside the healthcare environment, such as in the home or at an athletic facility. The participant will be allowed to phone the physiotherapist for further advice regarding their physical activities if needed.

### Ethical considerations

This study will be conducted in accordance with the declaration of Helsinki and with Swedish laws. The study protocol has been approved by the Ethics Committee at the Faculty of Health Sciences at Linköping University in Sweden (2010/1888-31 and 2011/262-32). Written informed consent will be obtained from all patients who are included in the study. Patients will be free to leave the study without explanation without any negative consequences on future treatment.

There are no known risks associated with patient participation in the study except for possible temporary muscle aches after exercise. All physiotherapists involved in the study must be registered at the National Board of Health and Welfare in Sweden. All personal patient details will be made anonymous before data-entry. There are no commercial interests tied to the study.

### Statistical methods and power calculation

A sample-size calculation suggested that that 60 patients/group was necessary to detect a 7% change in the NDI with a standard deviation for change of 13 with a 20% β-error (power 1- β = 080%) and an α-error of 0.05. We added an extra 20% of the estimated number for security, resulting in 72 patients/group and a total of 216 patients. Data will be analysed according to an intention-to-treat approach. An alternative analysis will be performed to take treatment compliance into consideration. Analyses will be performed with parametric or non-parametric statistics, depending on the type of data.

### Time frame

The estimated time period for patient inclusion in the study is 2 years. Follow-up will continue for another 2 years.

## Discussion

This is a prospective randomized, controlled, multi-centre trial with blinded assessors who are not involved in the treatment. In order to provide treatment in the participants’ home towns, several physiotherapists will be involved in the treatments. This is a disadvantage in that it will result in decreased control of treatment performance; however, all physiotherapists will undergo the same basic training for the study and will be carefully instructed by the project leaders. Both participants and physiotherapists will keep treatment diaries. This is also an advantage in that it will be possible to generalize the approach to primary health care conditions. Treatments will be individually tailored.

The Swedish Council on Technology Assessment in Health Care has stated that there is a scientific gap in knowledge in terms of the effects of exercise in adults with chronic pain [46]. NHS Evidence from the UK Database of Uncertainties about the Effects of Treatments (DUETs) has also identified both the effects of exercises for mechanical neck disorders (including WAD) [47] and the effects of conservative treatment for whiplash [48] to be important gaps in scientific knowledge. This study will help fill these gaps. The outcome of the study is expected to help improve clinical decision-making for WAD patients. The results will be directly applicable in clinical settings to improve the treatment of patients with chronic WAD.

## Conclusions

This study will provide practical knowledge about the effects of different exercise strategies on chronic WAD. It will help improve treatment guidelines, which must take into account both the patient’s physical and mental functioning and the cost-effectiveness of different strategies.

## Competing interests

The authors declare that they have no competing interests.

## Authors’ contributions

AP and GP initiated the study. AP, GP and MLL were responsible for the overall design of the study. TO, ÅD, LB, GJ and ASK are experts in their respective fields and critically discussed the study design with AP, GP and MLL. AP was as a project leader responsible for applying for funding. AP, GP and MLL were responsible for data collection. Data analysis will be performed by AP, GP and MLL with support from a statistician. AP, GP and MLL wrote the manuscript. All authors read, revised and approved the final manuscript.

## Pre-publication history

The pre-publication history for this paper can be accessed here:

http://www.biomedcentral.com/1471-2474/14/311/prepub

## References

[B1] The Whiplash Commission Final Report Sandviken 2005 [http://www.whiplashkommissionen.se/pdf/WK_finalreport.pdf]. Accessed March 4, 2012

[B2] Holm LW Carroll LJ Cassidy JD Hogg-Johnson S Côté P Guzman J Peloso P Nordin M Hurwitz E van der Velde G Carragee E Haldeman S The burden and determinants of neck pain in whiplash-associated disorders after traffic collisions: results of the bone and joint decade 2000–2010 task force on neck pain and its associated disorders Spine 2008 33 S52 S59 10.1097/BRS.0b013e3181643ece 18204401

[B3] Association of British Insurers Fair, independent, objective – ABI publishes proposals to curb the UK’s whiplash epidemic https://www.abi.org.uk/News/News-releases/2013/03/Fair-Independent-Objective--Abi-Publishes-Proposals-To-Curb-The-Uks-Whiplash-Epidemic. Accessed October 20, 2013

[B4] Styrke J Stålnacke BM Bylund PO Sojka P Björnstig U A 10-year incidence of acute whiplash injuries after road traffic crashes in a defined population in the northern Sweden Phys Med Rehabil 2012 4 739 747 10.1016/j.pmrj.2012.05.010 22819305

[B5] Cassidy JD Carroll LJ Côté P Effect of eliminating compensation for pain and suffering on the outcome of insurance claims for whiplash injury N Engl J Med 2000 342 1179 1186 10.1056/NEJM200004203421606 10770984

[B6] Rodriques AA Barr KP Burns SP Whiplash: pathophysiology, diagnosis, treatment and prognosis Muscle Nerve 2004 29 768 781 10.1002/mus.20060 15170609

[B7] Rebbeck T Sindhusake D Cameron ID Rubin G Feyer A-M Walsh J Gold M Schofield WN A prospective cohort study of health outcomes following whiplash associated disorders in an Australian population Inj Prev 2006 12 93 98 10.1136/ip.2005.010421 16595423 PMC2564458

[B8] Carroll L Holm L Hogg-Johnson S Cote P Cassidy D Haldeman S Nordin M Hurwitz E Carragee E van der Velde G Peloso P Guzman J Course and prognostic factors for neck pain in whiplash-associated disorders (WAD). Results of the bone and joint decade 2000–2010 task force on neck pain and its associated disorders Spine 2008 33 583 592 10.1097/BRS.0b013e3181643eb8 18204405

[B9] Leth-Petersen S Rotger GP Long-term labour-market performance of whiplash claimants J Health Econ 2009 28 996 1011 10.1016/j.jhealeco.2009.06.013 19683817

[B10] Spitzer WO Skovron ML Salmi LR Cassidy JD Duranceau J Suissa S Ziess E Scientific monograph of the Quebek task force on whiplash-associated disorders: redefining whiplash and its management Spine 1995 20 1S 73S 10.1097/00007632-199501000-00001 7604354

[B11] Sterner Y Gerdle B Acute and chronic whiplash disorders – a review J Rehab Med 2004 36 193 210 10.1080/16501970410030742 15626160

[B12] Seferiadis A Rosenfeld M Gunnarsson R A review of treatment interventions in whiplash-associated disorders Eur Spine J 2004 13 387 397 15133721 10.1007/s00586-004-0709-1 PMC3476583

[B13] Woodhouse A Vasseljen O Altered motor control patterns in whiplash and chronic neck pain BMC Musculoskel Disord 2008 9 1 10 10.1186/1471-2474-9-1 PMC2446396 18570647

[B14] Stewart MJ Maher CG Refshauge KM Randomized controlled trial of exercise for chronic whiplash-associated disorders Pain 2007 128 59 68 10.1016/j.pain.2006.08.030 17029788

[B15] Söderlund A Lindberg P Cognitive behavioural components in physiotherapy management of chronic whiplash associated disorders (WAD) – a randomized group study G Ital Med Lav Ergon 2007 29 A5 A11 17650736

[B16] Nijs J van Oosterwijck J De Hertogh W Rehabilitation of chronic whiplash: treatment of cervical dysfunctions or chronic pain syndrome? Clin Rheumatol 2009 28 243 251 10.1007/s10067-008-1083-x 19160000

[B17] Verhagen AP Scholten-Peeters G van Wijngaarden S de Bie RA Bierma-Zeinstra SMA Conservative treatment for whiplash Cochrane Database Syst Rev 2007 4 4 10.1002/14651858.CD003338.pub3 PMC8713438 17443525

[B18] Swedish Council on Health Technology Assessment (SBU) Back pain, Neck pain. An evidence based review. (In Swedish) (Ont i ryggen, ont i nacken: en evidensbaserad kunskapssammanställning). Nr 145/2, chapter 14–17 2000 Stockholm: SB Offsett AB

[B19] Bunketorp L Lindh M Carlsson J Stener-Victorin E The effectiveness of a supervised physical training model tailored to the individual needs of patients with whiplash-associated disorders – a randomized controlled trial Clin Rehabil 2006 20 201 217 10.1191/0269215506cr934oa 16634339

[B20] Vikne J Oedegaard A Laerum E Ihlebaek C Kirkesola G A randomized study of new sling exercise treatment vs traditional physiotherapy for patients with chronic whiplash-associated disorders with unsettled compensation claims J Rehab Med 2007 39 252 259 10.2340/16501977-0049 17468795

[B21] Jull G Sterling M Kenardy J Beller E Does the presence of sensory hypersensitivity influence outcomes of physical rehabilitation for chronic whiplash? A preliminary RCT Pain 2007 129 28 34 10.1016/j.pain.2006.09.030 17218057

[B22] Teasell RW McClure JA Walton D Pretty J Salter K Meyer M Sequeira K Death B A research synthesis of therapeutic interventions for whiplash-associated disorder (WAD): part 4 - noninvasive interventions for chronic WAD Pain Res Manag 2010 15 313 322 21038010 10.1155/2010/487279 PMC2975534

[B23] Carlsson AM Assessment of chronic pain. I. Aspects of the reliability and validity of the visual analogue scale Pain 1983 16 87 101 10.1016/0304-3959(83)90088-X 6602967

[B24] Vernon H Mior S The neck disability index: a study of reliability and validity J Manipulative Physiol Ther 1991 14 409 415 1834753

[B25] Peolsson A Hedlund R Ertzgaard S Öberg B Intra-and inter-tester reliability and age- and sex-specific range of motion of the neck Physiother Canada 2000 52 233 242

[B26] Wibault J Vaillant J Vuillerme N Dedering Å Peolsson A Using the cervical range of motion (CROM) device to assess head repositioning accuracy in individuals with cervical radiculopathy in comparison to neck healthy individuals Man Ther 2013 18 403 409 10.1016/j.math.2013.02.004 23473752

[B27] Capuano-Pucci D Rheault W Aukai J Bracke M Day R Patrick M Intratester and intertester, reliability of the cervical range of motion device Arch Phys Med Rehabil 1991 72 338 340 2009054

[B28] Peolsson A Almkvist C Dahlberg C Lindqvist S Pettersson S Age- and sex-specific reference values of a test of neck muscle endurance J Manipulative Physiol Ther 2007 30 171 177 10.1016/j.jmpt.2007.01.008 17416270

[B29] Peolsson A Hedlund R Öberg B Intra- and inter-tester reliability and reference values for hand strength J Rehab Med 2001 33 36 41 10.1080/165019701300006524 11480468

[B30] Kammerlind A-S Ledin T Ödkvist L Skargren E Influence of asymmetry of vestibular caloric response and age on balance and perceived symptoms after acute unilateral vestibular loss Clin Rehabil 2006 20 142 148 10.1191/0269215506cr886oa 16541934

[B31] Johansson G Jarnlo G-B Balance training in 70-year-old women Physiother Theory Pract 1991 7 121 125 10.3109/09593989109106962

[B32] Tait RC Chibnall JT Krause S The pain disability index: psychometric properties Pain 1990 40 171 182 10.1016/0304-3959(90)90068-O 2308763

[B33] Honrubia V Bell TS Harris MR Baloh RW Fisher LM Quantitative evaluation of dizziness characteristics and impact on quality of life Am J Otology 1996 17 595 602 8841705

[B34] Osman A Barrios FX Gutierrez PM Kopper BA Merrifield T Grittmann L The pain catastrophizing scale: further psychometric evaluation with adult samples J Behavioral Med 2000 23 351 365 10.1023/A:1005548801037 10984864

[B35] Stratford P Gill C Westaway M Binkley J Assessing disability and change on individual patients: a report of a patient specific measure Physiother Canada 1995 47 358 363

[B36] Altmaier ERD Kao C Lehmann T Weinstein J Role of self-efficacy in rehabilitation outcome among chronic low back pain patients J.Counsel Psychol 1993 36 725 733

[B37] Dzewaltowski D Toward a model of exercise motivation J Sport Exerc Psychol 1989 11 251 269

[B38] Craig CL Marshall AL Sjöström M Bauman AE Booth ML Ainsworth BE Pratt M Ekelund U Yngve A Sallis JF Oja P International physical activity questionnaire: 12-country reliability and validity Med Sci Sports Exerc 2003 35 1381 1395 10.1249/01.MSS.0000078924.61453.FB 12900694

[B39] Roelofs J Sluiter JK Frings-Dresen MH Goossens M Thibault P Boersma K Vlaeyen JW Fear of movement and (re)injury in chronic musculoskeletal pain: evidence for an invariant two-factor model of the Tampa scale for Kinesiophobia across pain diagnoses and Dutch, Swedish, and Canadian samples Pain 2007 131 181 190 10.1016/j.pain.2007.01.008 17317011

[B40] Zigmond AS Snaith RP The hospital anxiety and depression scale Acta Psychiatr Scand 1983 67 361 370 10.1111/j.1600-0447.1983.tb09716.x 6880820

[B41] Brooks RG Jendteg S Lindgren B Persson U Björk S EuroQol: health-related quality of life measurement. Results of the Swedish questionnaire exercise Health Policy 1991 18 37 48 10.1016/0168-8510(91)90142-K 10112300

[B42] Sullivan M Karlsson J Ware J The Swedish SF-36 health survey-I. Evaluation of data quality, scaling assumptions, reliability and construct validity across general populations in Sweden Soc Sci Med 1995 41 1349 1358 10.1016/0277-9536(95)00125-Q 8560302

[B43] de Zwart BC Frings-Dresen MH van Duivenbooden JC Test-retest reliability of the work ability index questionnaire Occup Med 2002 52 177 181 10.1093/occmed/52.4.177 12091582

[B44] Siegrist J Starke D Chandola T Godin I Marmot M Niedhammer I Peter R The measurement of effort-reward imbalance at work: European comparisons Soc Sci Med 2004 58 1483 1499 10.1016/S0277-9536(03)00351-4 14759692

[B45] Howie JG Heaney DJ Maxwell M Walker JJ A comparison of a patient enablement instrument (PEI) against two established satisfaction scales as an outcome measure of primary care consultations Fam Pract 1998 15 165 171 10.1093/fampra/15.2.165 9613486

[B46] Swedish Council on Health Technology Assessment (SBU’s) assignment on treatment uncertainties http://www.sbu.se/sv/Publicerat/Sok-kunskapsluckor/Kunskapsluckor/Fysisk-traning-inom-halso--och-sjukvardens-ram-vid-otillracklig-fysisk-aktivitet-hos-vuxen-med-kronisk-smarta/

[B47] NHS Evidence from the UK Database of Uncertainties about the Effects of Treatments (DUETs) http://www.library.nhs.uk/duets/ViewResource.aspx?resID?=?412800&tabID?=?297&catID=. 14509

[B48] NHS Evidence from the UK Database of Uncertainties about the Effects of Treatments (DUETs) http://www.library.nhs.uk/duets/ViewResource.aspx?resID?=?332541&tabID?=?297

